# Numerical simulation study on the propagation and attenuation of shock waves in ventilation tunnels

**DOI:** 10.1038/s41598-025-29039-6

**Published:** 2025-11-26

**Authors:** Wei Li

**Affiliations:** 1 CCTEG China Coal Research Institute Co., Ltd, Beijing, 100013 China; 2 CCRI Tongan (Beijing) Intelligent Control Technology Co., Ltd, Beijing, 100013 China; 3 State Key Laboratory of Intelligent Coal Mining and Strate Control, Beijing, 100013 China

**Keywords:** Gas explosion, Bifurcation tunnel, Shock wave, Attenuation coefficient, Numerical simulation, Engineering, Physics

## Abstract

This study systematically investigated the influence of tunnel spatial characteristics on the propagation and attenuation patterns of gas explosion shock waves by establishing models of straight tunnels with varying bending angles and branched tunnels with different branching angles. Results indicate: In single tunnels, increasing curvature angle induces a multi-peak pressure distribution, with peak pressure first rising, then decreasing, and subsequently recovering. A distinct inflection point occurs at a 60° curvature angle. For branched tunnels, when the branching angle is 30° or 45°, the first pressure peak within the branch tunnel increases with distance. However, when the branching angle is 60°, 120°, 135°, or 150°, the first pressure peak first decreases and then increases with distance. Additionally, the attenuation coefficient of the first pressure peak in bifurcated tunnels decreases initially and then increases with increasing bifurcation angle, reaching its minimum around 60°. Although the rate of decrease in explosion pressure within horizontal tunnels diminishes as the bifurcation angle increases, shock wave attenuation in bifurcated tunnels remains overall greater than in straight tunnels. These results reveal the significant influence of tunnel geometry on explosive dynamic response, providing quantitative evidence for optimizing mine tunnel design and enhancing blast resistance safety.

## Introduction

Gas explosions are highly destructive disasters that frequently occur in coal mines^1,2^. Coal mine gas, specifically methane, can rapidly combust and cause an explosion when its concentration reaches the explosive limit and encounters an ignition source, producing a violent shock wave^3,4^. This shock wave carries immense energy, propagates rapidly, and poses serious threats to the safety of mine structures, equipment, and personnel^5,6^.

The basic theory of shock wave propagation reveals the laws of wave transmission in different media and structures^7–9^. The propagation of shock waves in tunnels involves not only gas dynamics but is also significantly influenced by the geometric structure of the tunnels^10,11^. Single tunnels are the most common structure in mines, and their relatively simple linear geometry facilitates the study of shock wave propagation laws^12,13^. However, the mine tunnel systems are often more complex, with many bifurcation tunnels, making the propagation paths of shock waves complex and variable^14^. For gas explosion shock wave propagation, numerous theoretical and experimental studies have been conducted by scholars at home and abroad. Jing et al.. established the tunnel gas explosion model^15,16^. The influences of the opening and closing of the branch tunnel on the length and angle of the branch tunnel^17^ The gas explosion in the tunnel forms a reflection effect at the corner, and the peak pressure at the corner is about twice higher than that of the straight pipe^18^. The coupling explosion of gas and coal dust in the branch pipe, the pressure mutation coefficient gradually increases with the increase of the bifurcation angle of the pipe^19^. Through physical experiments, the peak value of the shock wave of the explosion in the pipeline is not affected by the length of the pipeline^20^. The overpressure of the explosion wave and the flame propagation velocity in the bifurcated pipeline are also studied^21^. In bifurcation tunnels, shock waves undergo reflection, refraction, and diffraction during propagation, leading to complex changes in energy distribution and propagation paths^22,23^.

Currently, research on the bending angles of methane explosions in single and branched mine tunnels lacks systematic and comprehensive approaches. Particularly, there remains insufficient understanding of shock wave propagation patterns influenced by the complex geometric structures of real mine tunnels. Existing studies predominantly focus on simplified pipeline conditions for methane explosions, making it difficult to systematically summarize universal principles applicable to underground coal mines. Therefore, this study focuses on the unique geometric configurations of mine tunnels with varying bending and branching angles. By establishing physical models that accurately represent actual tunnel topologies, systematically simulating and comparing the propagation and attenuation behavior of shock waves within complex tunnel networks. This bridges the research gap between “pipe” and “tunnel” scales and between idealized conditions and real geological structures, providing direct guidance for blast-resistant design and safety protection in mine tunnels. The findings hold significant engineering reference value.

## Numerical simulation effectiveness study

### Fundamental theory of numerical simulation

The explosion of combustible gases in a confined space is essentially a rapid combustion reaction process that satisfies the conservation of mass, momentum, energy, and chemical composition balance. The simplified equations for numerical simulation of combustible gas explosions are as follows^24,25^.

Mass conservation equation:

1 $$\frac{{\partial \rho }}{{\partial t}}+\frac{{\partial (\rho {u_j})}}{{\partial {x_j}}}=0$$

Momentum conservation equation:

2 $$\ \begin{gathered} \frac{{\partial \left( {\rho \mu _{i} } \right)}}{{\partial t}} + \frac{{\partial \left( {\rho \mu _{i} \mu _{j} } \right)}}{{\partial x_{j} }} = - \frac{{\partial p}}{{\partial x_{i} }} + \frac{\partial }{{\partial x_{j} }} \hfill \\ \left[ {\left( {\mu + \mu _{t} } \right)\left( {\frac{{\partial \mu _{i} }}{{\partial x_{j} }} + \frac{{\partial \mu _{j} }}{{\partial x_{i} }} - \frac{2}{3}\delta _{{ij}} \frac{{\partial \mu _{k} }}{{\partial x_{k} }}} \right)} \right] \hfill \\ \end{gathered}$$

Energy conservation equation:

3 $$\frac{{\partial \left( {\rho h} \right)}}{{\partial t}} + \frac{{\partial \left( {\rho \mu _{j} h} \right)}}{{\partial x_{j} }} = \frac{\partial }{{\partial x_{j} }}\left[ {\left( {\frac{\mu }{{\sigma x_{j} }} + \frac{{\mu _{t} }}{{\sigma _{{ht}} }}} \right)\frac{{\partial h}}{{\partial x_{j} }}} \right] + \frac{{Dp}}{{Dt}} + S_{h}$$

The total enthalpy is defined as:

4 $$h = c_{p} T + \frac{1}{2}u_{i} u_{i}$$

Chemical composition balance equation:

5 $$\frac{{\partial \left( {\rho Y_{{fu}} } \right)}}{{\partial t}} + \frac{{\partial \left( {\rho u_{j} Y_{{fu}} } \right)}}{{\partial x_{j} }} = \frac{\partial }{{\partial x_{j} }}\left[ {\left( {\frac{\mu }{{\sigma _{Y} }} + \frac{{\mu _{t} }}{{\sigma _{{Y,t}} }}} \right)\frac{{\partial Y_{{fu}} }}{{\partial x_{j} }}} \right] + R_{{fu}}$$

Turbulence Model (Standard $k - \varepsilon$ Model)

Turbulent Kinetic Energy Equation *k*:

6 $$\frac{{\partial \left( {\rho \varepsilon } \right)}}{{\partial t}} + \frac{{\partial \left( {\rho u_{j} k} \right)}}{{\partial x_{j} }} = \frac{\partial }{{\partial x_{j} }}\left[ {\left( {\mu + \frac{\mu }{{\sigma _{k} }}} \right)\frac{{\partial k}}{{\partial x_{j} }}} \right] + P_{k} - \rho \varepsilon$$

Turbulent Dissipation Rate Equation ε:

7 $$\frac{{\partial \left( {\rho \varepsilon } \right)}}{{\partial t}} + \frac{{\partial \left( {\rho u_{j} k} \right)}}{{\partial x_{j} }} = \frac{\partial }{{\partial x_{j} }}\left[ {\left( {\mu + \frac{\mu }{{\sigma _{k} }}} \right)\frac{{\partial k}}{{\partial x_{j} }}} \right] + C_{{\varepsilon 1}} \frac{\varepsilon }{k}P_{k} \_C_{{\varepsilon 2}} \rho \frac{{\varepsilon ^{2} }}{k}$$

Where: ρ is density; u_i_, u_j_, u_k_ are velocity components; p is pressure; μ is laminar flow dynamic viscosity; μ_t_ is turbulent dynamic viscosity; h is total enthalpy; c_p_ is specific heat capacity; *T* is temperature; Y_fu_ is the mass fraction of fuel; R_fu_ is the chemical reaction rate; *k* is turbulent kinetic energy; ε is turbulent dissipation rate; $\sigma _{k} ,\,\sigma _{\varepsilon } ,\,\sigma _{h} ,\,\sigma _{y}$ are turbulence prandtl number; $C_{\mu } ,\,C_{{\varepsilon 1}} ,\,C_{{\varepsilon 2}}$ turbulence model constants; $\delta _{{ij}}$ Kronecker delta.

The tunnel walls are defined as isothermal, smooth, and non-slip boundaries to simulate the physical properties of actual wellbore walls. One end of the tunnel is closed, while the other end is set as a pressure outlet or free-flowing open boundary to simulate the outward dispersion of explosion products. The air domain boundary is set under pressure far-field conditions with an initial pressure of 100 kPa and temperature of 298 K. The ignition source is triggered by high-voltage discharge with an initial energy of 10 J, and a 9.5% methane-air premixed gas is introduced via local initialization. Additionally, to minimize numerical reflections, a sufficiently large air domain is established at the open boundary to prevent non-physical reflections.

### Numerical method effectiveness verification

#### Grid independence verification

**Fig. 1:**
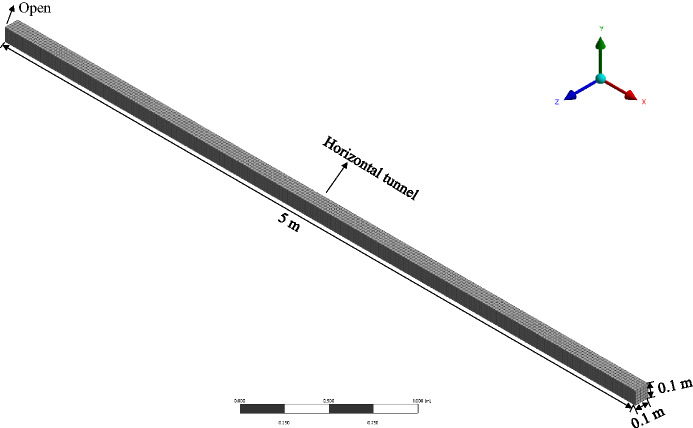
Model diagram.

**Fig. 2:**
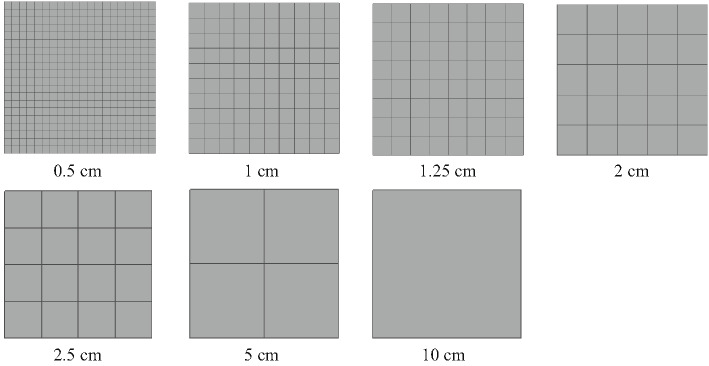
Different grid sizes diagram.

The size of the grid has a certain impact on the simulation results. To determine the appropriate grid size arrangement, this study used seven sets of grid sizes of 0.5 cm, 1 cm, 1.25 cm, 2 cm, 2.5 cm, 5 cm, and 10 cm for verification. The simulation verification object is a horizontal tunnel model with dimensions of 5 m × 0.1 m × 0.1 m, with all surfaces set as solid boundaries except for the right side, which is set as an open state. The horizontal tunnel is filled with a methane/air premix with a concentration of 9.5%^26^. The ignition source is set at the geometric center of the closed end on the left side^27^, 0.1 m from the closed end on the left side and 0.25 m from the upper wall. The physical model and grid size in the simulation process are shown in Figs. 1 and 2.

To obtain the appropriate grid size, the explosion pressure peak values at each measuring point under different grid sizes were detected, with the ignition source located 0.1 m from the left wall. Table 1 shows the comparison results of the explosion pressure peak values obtained at 0.7 m (P_1_), 1.5 m (P_2_), and 3.1 m (P_3_) from the ignition source with seven sets of grid sizes. As can be seen from Table 1, as the grid size increases, the absolute error and relative error averages of P_1_, P_2_, and P_3_ between the simulated values and experimental values first decrease and then increase. When the grid size is 2 cm, the average absolute error and relative error between the simulated values and experimental values are the smallest, at 1.54 kPa and 1.29%, respectively, indicating that when the grid size is 2 cm, the simulation results are highly consistent with the experimental results.

**Table 1:** Comparison of explosion pressure peak values between simulation and experiment under different grid sizes.

Grid size /cm	*P*_1_ experimental value /kPa	*P*_1_ simulated value /kPa	*P*_2_ experimental value /kPa	*P*_2_ simulated value /kPa	*P*_3_ experimental value /kPa	*P*_3_ simulated value /kPa	Mean absolute error /kPa	Mean relative error /%
0.5	123.67	119.87	123	115.24	116.49	108.83	6.41	5.60
1	122.27	121.71	120.69	120.87	119.2	110.46	3.16	2.69
1.25	120.6	120.82	122.71	120.22	117.03	112.59	2.38	2.02
2	121.04	120.33	122.43	123.45	116.67	113.78	1.54	1.29
2.5	120.06	119.98	121.29	119.4	119.67	113.75	2.63	2.24
5	121.09	113.57	123.35	114.67	116.36	110.94	7.21	6.38
10	123.51	111.12	123.26	111.06	117.95	109.03	11.17	10.12

Additionally, to quantify the grid discretization error and further validate grid convergence, this study employs the Grid Convergence Index (GCI) method for supplementary analysis^28^. The GCI provides an estimate of the discretization error based on the Richardson extrapolation method, serving as a standard approach for assessing the convergence of numerical simulations.Taking a sequence of three grids-coarse (5 cm), medium (2.5 cm), and fine (2 cm)-as an example, the peak pressure at measurement point P1 was selected as the key variable for calculation. The resulting GCI values are shown in the table below.

**Table 2:** Grid convergence index analysis for peak pressure at P_1_.

Grid Size (cm)	Peak Pressure (KPa)	GCL < sub > fine</sub> (%)	GCL < sub > medium</sub> (%)
5.0(coarse)	113.57	-	-
2.5(medium)	119.98	3.21	-
2.0(fine)	120.33	-	0.29

The computational results indicate that the GCl < sub > medium</sub > value from medium to fine mesh is only 0.29%, significantly smaller than the GCI < sub > fine</sub > value from medium to coarse mesh. This indicates that the solution has entered the regime of asymptotic convergence, and selecting a finer mesh (e.g., 1.25 cm) would have negligible impact on the results. Therefore, the 2 cm mesh size achieves an optimal balance between computational accuracy and efficiency, yielding a mesh-independent numerical solution.

#### Calculation domain size verification

**Fig. 3:**
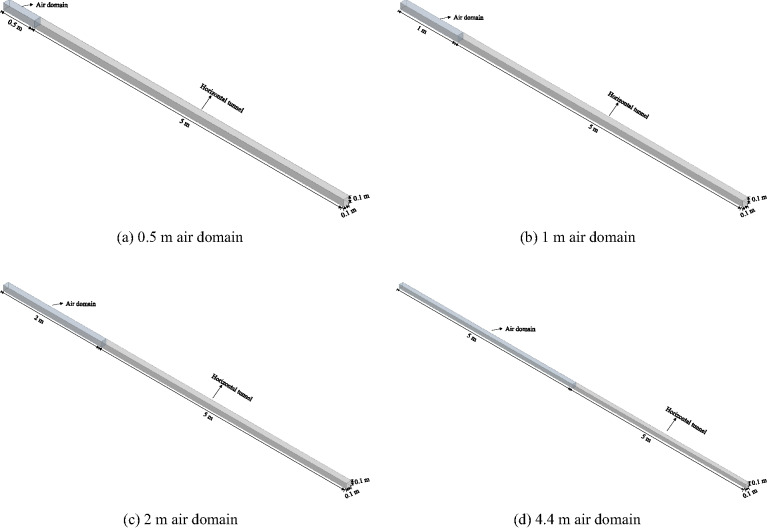
Different sizes of air domains. (**a**) 0.5 m air domain, (**b**) 1 m air domain, (**c**) 2 m air domain, (**d**) 4.4 m air domain.

The air domain may affect the results of numerical simulations^29^. By setting the right end of the horizontal tunnel as an opening and the other surfaces as adiabatic walls, an air domain was set on the right side of the horizontal tunnel. Four different air domain sizes of 0.5 m × 0.1 m × 0.1 m, 1 m × 0.1 m × 0.1 m, 2 m × 0.1 m × 0.1 m, and 5 m × 0.1 m × 0.1 m were selected for verification, as shown in Fig. 3. Although the results in Table 2 indicate that the size of the air domain has minimal impact on the simulated pressure peaks, a larger air domain (4.4 m in this study) was adopted in subsequent simulations to ensure numerical stability and to minimize potential wave reflections from the open boundary, which could otherwise introduce unphysical oscillations in the flow field.

Table 3 shows the comparison results of the explosion pressure peak values obtained at 0.7 m (P’1), 1.5 m (P’2), and 3.1 m (P’3) from the interface between the air domain and the horizontal tunnel under four different air domain sizes. As can be seen from Table 2, different air domain sizes have minimal differences in the average absolute error and average relative error of the explosion pressure peak values between the simulation and experiment at each measuring point. The average absolute error and average relative error are approximately 2.42 kPa and 2.05%, respectively, indicating that the size of the air domain does not affect the calculation results.

**Table 3:** Comparison of explosion pressure peak values between simulation and experiment under different air domain sizes.

Air domain size /m	*P*’_1_ experimental value /kPa	*P*’_1_ simulated value /kPa	*P*’_2_ experimental value /kPa	*P*’_2_ simulated value /kPa	*P*’_3_ experimental value /kPa	*P*’_3_ simulated value /kPa	Mean absolute error /kPa	Mean relative error /%
0.5	123.75	120.08	124.37	120.53	118.26	117.16	2.87	2.41
1	121.17	118.5	121.7	120.23	117.3	114.64	2.27	1.93
2	120.17	120.9	123.7	117.71	115.89	116.75	2.53	2.13
4.4	120.17	118.38	121.07	120.37	118.36	114.76	2.03	1.72

#### Experimental comparison verification

**Fig. 4:**
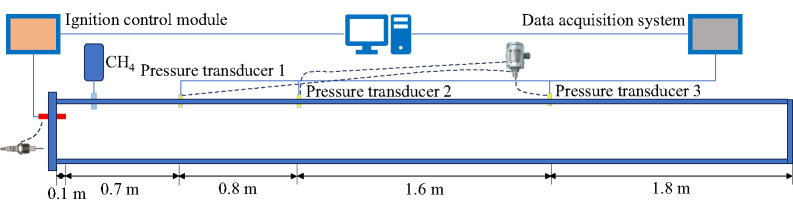
Simplified diagram of the experimental apparatus.

To ensure the reliability of the numerical method, the numerical simulation results were compared with laboratory experiments. The simplified diagram of the experimental apparatus is shown in Fig. 4. The experimental apparatus is 5 m long, with a cross-sectional size of 0.1 m × 0.1 m, and a wall thickness of 0.01 m, with a maximum pressure resistance of about 3000 kPa. During the experiment, a PMC13 piezoelectric pressure sensor (measuring range − 100 kPa to 2000 kPa, accuracy 1 kPa, pressure sampling interval 0.2 ms) was used. The left side of the horizontal tunnel was set as a closed state, and the right side was set as an open state. The tunnel was filled with methane at a concentration of 9.5%, and the ignition source was set at a position 10 cm from the left wall, ignited by high-voltage discharge with an ignition energy of 10 J. Three explosion pressure sensors were set on the upper wall of the horizontal tunnel at distances of 0.7 m, 1.5 m, and 3.1 m from the ignition source, referred to as measuring points 1, 2, and 3, i.e., P_1_, P_2_, and P_3_. To ensure the reliability and reproducibility of the experimental data, each test condition was repeated three times. The average values of the peak pressures were used for comparison with the numerical simulation results. The standard deviations of the peak pressures at measuring points P_1_, P_2_, and P_3_ were 2.1 KPa, 1.8 KPa, and 2.5 KPa, respectively, indicating good consistency among repeated experiments.

**Fig. 5:**
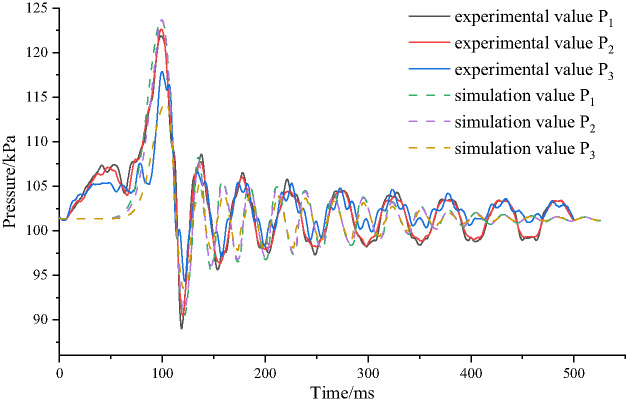
Comparison of pressure time histories between numerical simulation and experiment.

In this model, the methane concentration, environmental parameters, tunnel size, and measuring point positions were consistent with the experimental apparatus. The tunnel walls were set as adiabatic and smooth, and the grid size was set to 2 cm. The numerical simulation results were compared with the experimental data, as shown in Fig. 5.

Figure 5 shows that the trends in the explosion pressure time histories at measuring points 1 to 3 measured by the numerical simulation and the experiment are relatively consistent, with the explosion pressure first increasing, then decreasing, and subsequently oscillating repeatedly. Table 4 shows the comparison of peak values at measuring points 1, 2, and 3. The absolute error and relative error of measuring points 1 and 3 are larger than those of measuring point 2, but the trend of the numerical simulation data and experimental data is generally consistent. The analysis indicates that the errors between the numerical simulation data and experimental data are mainly due to factors such as the accuracy and sensitivity of the pressure sensor (PMC13), heat loss from the walls, and wall roughness^30^.

**Table 4:** Comparison of peak explosion pressure values between numerical simulation and experiment.

Point	experimental value/kPa	Simulation value/kPa	Absolute error /kPa	Relative error /%	Mean absolute error /kPa	Mean relative error /%
1	121.57	124.42	2.85	2.29	2.37	2
2	123.54	124.36	0.82	0.66		
3	116.49	113.04	3.45	3.05		

## Shock wave propagation and attenuation study

### Shock wave propagation in single tunnels

#### Physical model

**Fig. 6:**
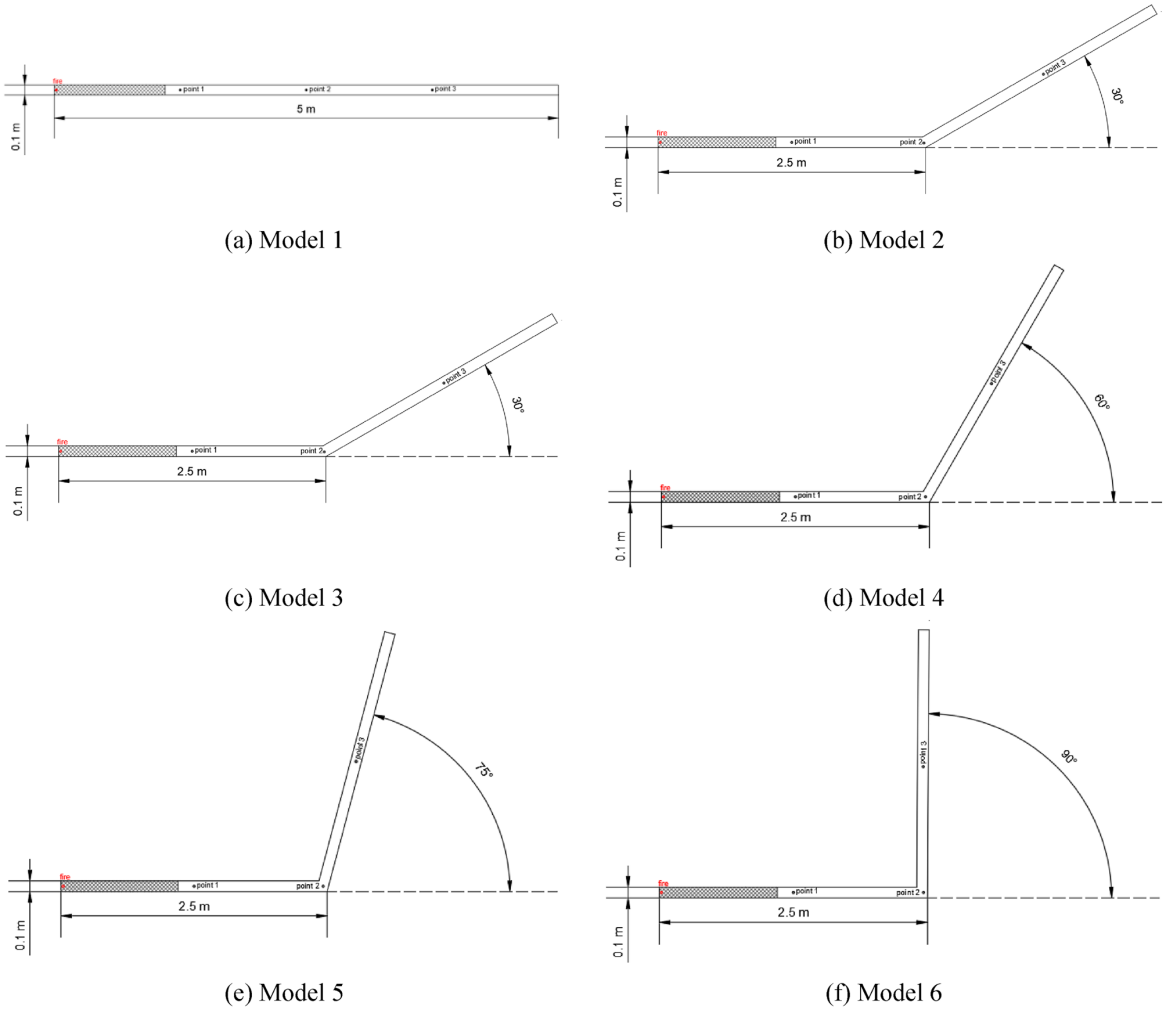
Physical models of single tunnels with different bending angles. (**a**) Model 1, (**b**) Model 2, (**c**) Model 3, (**d**) Model 4, (**e**) Model 5, (**f**) Model 6.

As shown in Fig. 6, Model 1 consists of a rectangular tunnel with a length of 5 m and a cross-sectional size of 0.1 m × 0.1 m. All walls are set as smooth adiabatic walls except for the right side, which is set as an opening. The ignition source is located 0.01 m from the left end, 0.075 m from the bottom of the tunnel, and 0.05 m from the side. Three measuring points (measuring points 1 to 3) are located on the centerline of the horizontal tunnel at distances of 1.25 m, 2.5 m, and 3.75 m from the left end, respectively. The shaded area is filled with methane at a concentration of 9.5%. Models 2 to 6 are numerical models with bending angles of 30°, 45°, 60°, 75°, and 90°, respectively, with the same calculation parameters as Model 1.

#### Results analysis

1. The influence of tunnel bending angle on gas explosion pressure

To study the influence of tunnel bending angle on gas explosion pressure, the distribution of gas explosion pressure at bending angles of 30°, 45°, 60°, 75°, and 90° was compared based on a horizontal tunnel gas explosion.

**Fig. 7:**
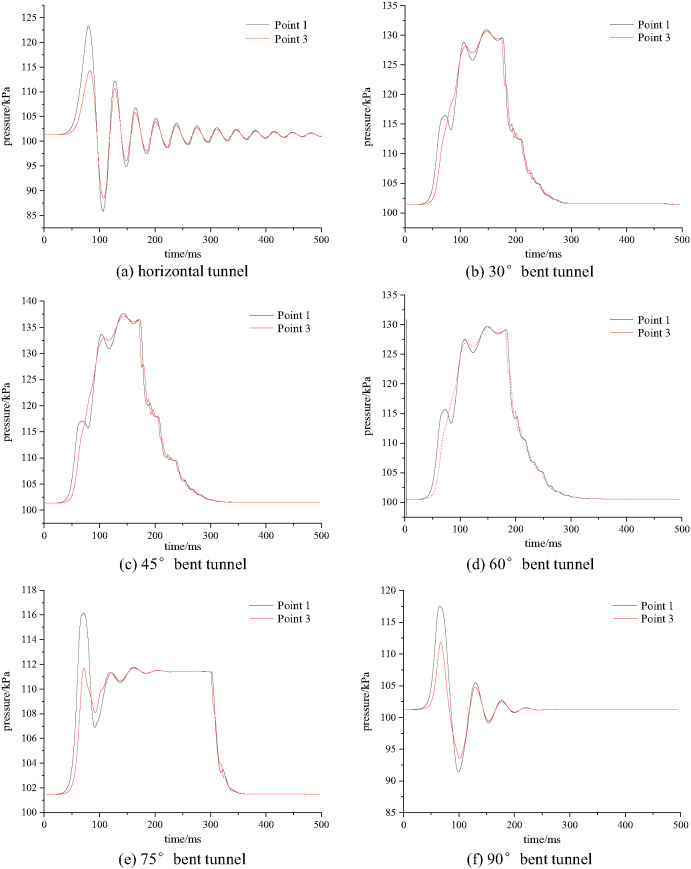
Explosion time histories under different bending angles.

As shown in Fig. 7, as the angle of the horizontal tunnel increases, each measuring point in the tunnel first shows a multi-peak trend, followed by a larger peak value, with the peak pressure showing an initial increase, then a decrease, followed by another increase. When the bifurcation tunnel angle is less than 60°, the contact area between combustible gas and air at the bend is larger, promoting the propagation of explosion pressure, and explosion energy is more easily accumulated inside the horizontal tunnel. Therefore, compared with the horizontal tunnel, the explosion pressure peak value inside the acute angle tunnel increases.

As the angle increases, the tunnel wall impedes the outward leakage of explosion energy, causing energy to accumulate again and thereby elevating the peak explosion pressure value. The primary physical mechanisms include: Flow separation and vortex formation: At high bending angles, due to flow inertia, high-velocity combustion products and shock waves cannot adhere to the tunnel wall, leading to significant flow separation at the inner bend angle. This separation generates a recirculation zone or vortex that acts as a temporary barrier, increasing flow resistance and causing upstream pressure buildup. Wall-induced pressure accumulation also contributes to peak pressure increases, as the explosion wave impinges nearly perpendicularly onto the curved wall surface. This direct impact generates a strong “splash” effect. The confined geometry prevents rapid downstream pressure release, resulting in significant pressure buildup upstream of the bend and at measurement points within these regions.

(2) The influence of tunnel bending angle on explosion pressure contour

**Fig. 8:**
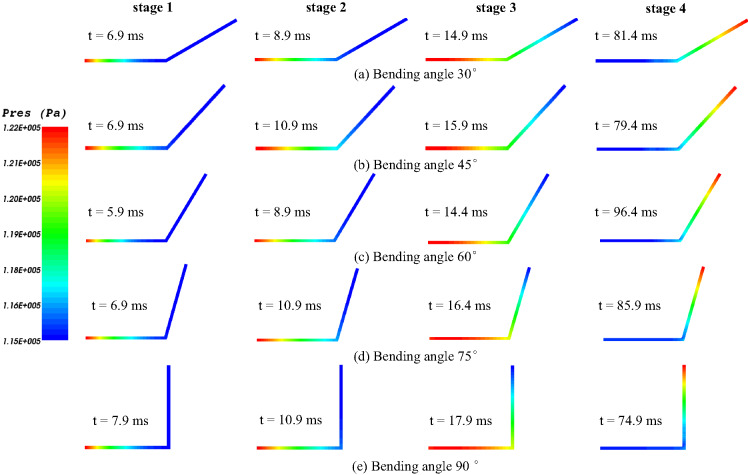
Explosion pressure contours under different bending angles.

By simulating the explosion pressure contours at different bending angles, four stages of the gas explosion process in the tunnel were selected, as shown in Fig. 8. In Stage 1, the propagation distance of explosion pressure increases with the increase in bending angle, but the increase in propagation distance is relatively small. In Stage 2, the pressure propagates to the bending angle for bending angles greater than 30°. In Stage 2, the propagation distance of explosion pressure increases significantly with the increase in bending angle. In Stage 4, the bending tunnel reduces the attenuation of gas explosion pressure, and the larger the bending angle, the slower the attenuation of gas explosion.

(3) Attenuation coefficient analysis

**Fig. 9:**
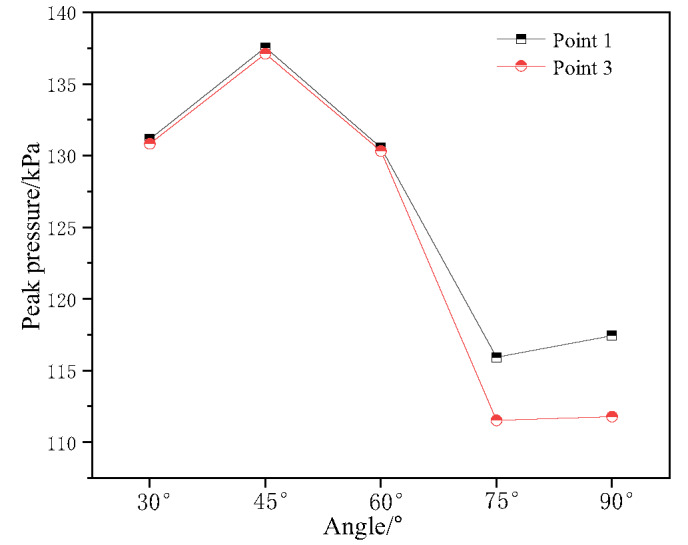
Comparison of explosion pressure peak values under different angles.

Using measuring points 1 and 3 in the tunnel as research objects, the explosion pressure peak value curves at the measuring points in the tunnel were plotted, as shown in Fig. 9.

As shown in Fig. 9, as the bifurcation tunnel angle increases, the explosion pressure peak values at measuring points 1 and 3 first increase, then decrease, and then increase again. The difference in explosion pressure peak values between measuring points 1 and 3 gradually increases. When the bifurcation tunnel angle is between 30° and 60°, the contact area between combustible gas and air at the bend is larger, promoting the propagation of explosion pressure, and explosion energy is more easily accumulated inside the horizontal tunnel. Therefore, compared with the horizontal tunnel, the explosion pressure peak value inside the acute angle tunnel increases. As the angle increases, the chemical reaction inside the horizontal tunnel becomes more sufficient, causing the explosion pressure to attenuate during the subsequent propagation due to a lack of energy supply. When the bifurcation tunnel angle is 90°, the obstruction effect of the tunnel walls is stronger than that of the bifurcation tunnel at 75°, resulting in an increase in explosion pressure peak value. This is attributed to the mechanisms of wave impingement and confined combustion: The near-perpendicular impact of the shock front on the bend wall creates a localized high-pressure zone due to the stagnation of flow. Furthermore, the confinement restricts the expansion of combustion products, effectively increasing their density and pressure in the region preceding the bend. The enhanced turbulence also promotes more complete combustion near the bend, contributing to the observed pressure rise.

In tunnels, the attenuation of explosion pressure is usually described by the attenuation coefficient. The attenuation coefficient is a parameter that indicates the degree of pressure attenuation with distance. The attenuation of explosion pressure can be affected by various factors, such as tunnel geometry, explosion source characteristics, and the properties of the gas in the tunnel. The general expression of explosion pressure attenuation is as follows^31^,

8 $${P_{\left( x \right)}}={P_0} \cdot {e^{ - \alpha x}}$$

Where: *P*_(*x*)_ is the pressure at a distance x from the explosion source; *P*_0_ is the initial pressure at the explosion source; *α* is the attenuation coefficient; *x* is the distance from the explosion source.

After transformation, the explosion pressure attenuation coefficient can be expressed as^32^,

9 $$\alpha =\frac{{\ln {P_0} - \ln {P_{\left( x \right)}}}}{x}$$

**Fig. 10:**
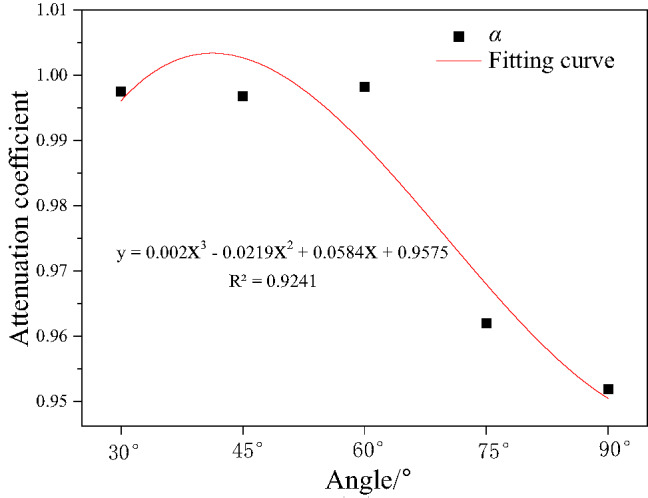
Attenuation coefficients under different bending angles.

Taking the explosion pressure at measuring point 1 as the initial value, i.e., x is 2.5 m, the explosion pressure attenuation coefficients at different tunnel bending angles were calculated, as shown in Fig. 10. As the bifurcation tunnel angle increases, the attenuation of explosion pressure inside the tunnel increases, and the larger the angle, the greater the attenuation value of explosion pressure inside the bifurcation tunnel. This indicates that the bifurcation tunnel angle is related to the attenuation coefficient of explosion pressure inside the tunnel, with the explosion pressure first increasing rapidly and then gradually decreasing.

(4) The influence of gas filling volume on explosion pressure time history in tunnels.

**Fig. 11:**
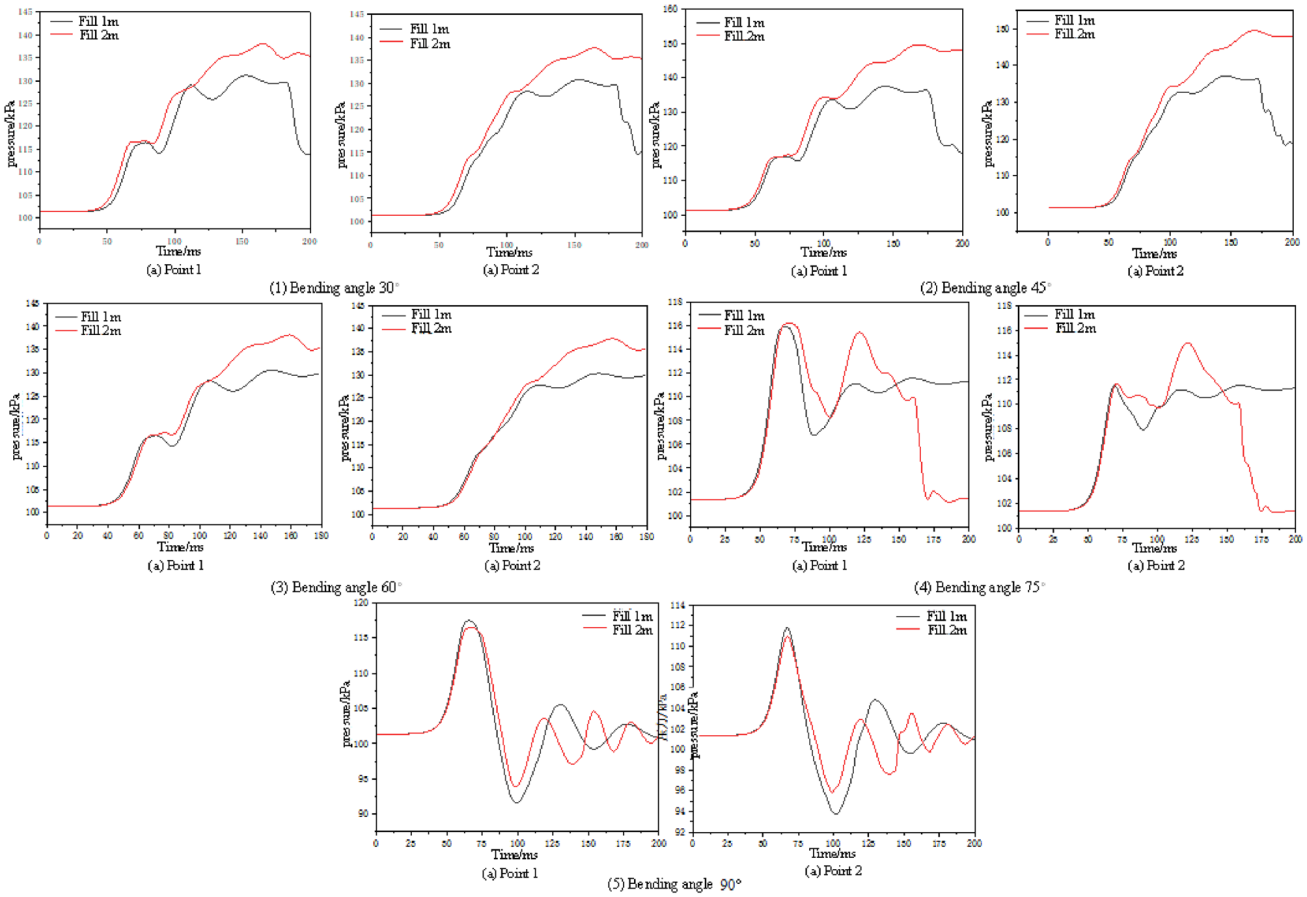
Comparison of explosion pressure time histories at different angles.

To study the impact of gas filling volume on explosion pressure inside the tunnel, the gas filling volume was changed, and the explosion pressure time history trends at measuring points 1 and 2 were compared for gas filling volumes of 1 m and 2 m, as shown in Fig. 11.

As shown in Fig. 11, as the bifurcation tunnel angle increases, the explosion pressure peak values at each measuring point for a gas filling volume of 1 m are initially smaller than the explosion pressure peak values at each measuring point for a gas filling volume of 2 m, and then become smaller than the explosion pressure peak values at each measuring point for a gas filling volume of 2 m. When the intersection angle is 75°, After 150 milliseconds, the differing trends in explosion pressure values between the 2-meter and 1-meter gas injection lengths primarily stem from variations in the total volume of combustible gas and the scale of energy release: The 2-meter injection length provides a greater volume of premixed gas, resulting in a longer explosion duration and more complete combustion. Consequently, it maintains a higher pressure level in the later stages due to reflected wave superposition, even exhibiting a pressure rebound. In contrast, the 1-meter refueling length involves a smaller total gas volume and lower energy release scale, resulting in a lower peak explosion pressure that decays more rapidly. By 150 milliseconds, it has entered a stable decay phase with continuous pressure reduction. Additionally, the tunnel’s curvature angle causes variations in pressure wave reflection, superposition, and turbulence effects, further amplifying the differences in pressure propagation and decay behavior between the two refueling lengths.

### Shock wave propagation in bifurcated tunnels

#### Physical model

**Fig. 12:**
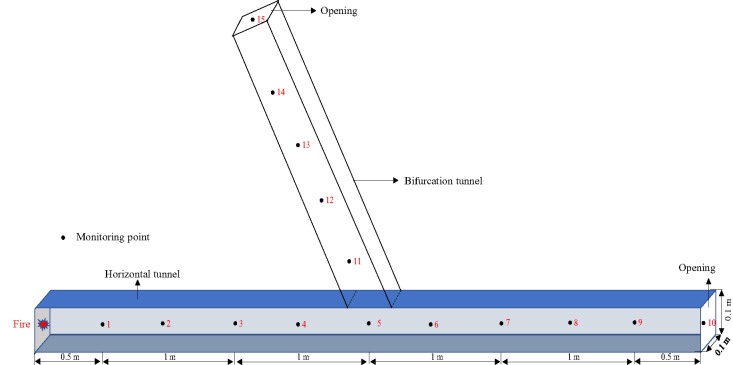
Physical model of the 120° bifurcation tunnel.

To further analyze the impact of gas explosions and tunnel bending angles on the propagation in horizontal tunnels, the tunnels were filled with gas, and physical models with bifurcation angles of 30°, 45°, 60°, 90°, 120°, 135°, and 150° were established. The schematic diagram of the physical model is shown in Fig. 12, and the origin of the model coordinates is set to (– 2.5, – 0.05, – 0.05). The open faces of the tunnel physical models are set as free-flowing open boundaries, with an initial pressure value of 100 kPa and an initial temperature of 298 K^33^. All bifurcation tunnel models share the same geometric dimensions, including the main tunnel length of 5 m, a cross-sectional size of 0.1 m × 0.1 m, and consistent bifurcation segment lengths. The origin of the model coordinates is set at (– 2.5, – 0.05, – 0.05) for all cases to ensure uniformity across different bifurcation angles.

#### Results analysis

1. Attenuation law of gas explosion in bifurcation tunnels

**Fig. 13:**
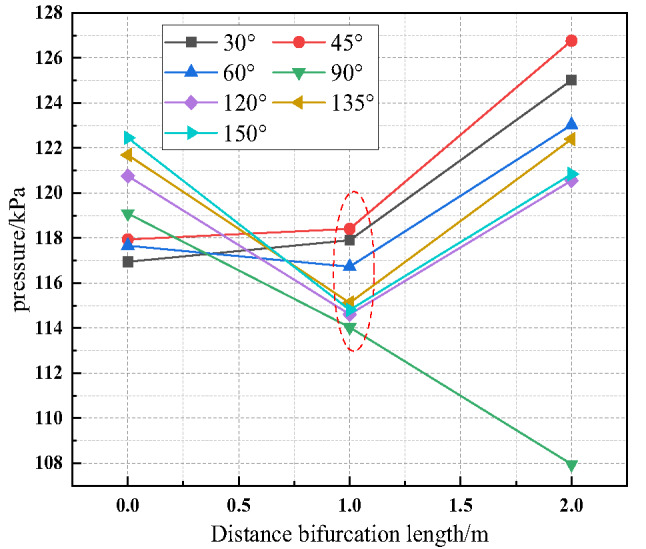
Variation of the first pressure peak with distance at different bifurcation angles.

Using measuring points 3, 6, and 7 in the bifurcation tunnel as research objects, the variation trends of the first pressure peak value of methane-air explosions in the bifurcation tunnel at different bifurcation angles (30°, 45°, 60°, 90°, 120°, 135°, 150°) were obtained, as shown in Fig. 13.

Figure 13indicates that when the bifurcation tunnel angle is 30° or 45°, the variation trend of the first pressure peak value in the bifurcation tunnel is generally similar, with the first pressure peak value of the methane-air explosion inside the bifurcation tunnel increasing with distance. When the bifurcation tunnel angle is 60°, 120°, 135°, or 150°, the first pressure peak value of the methane-air explosion inside the bifurcation tunnel first decreases and then increases with distance.

**Fig. 14:**
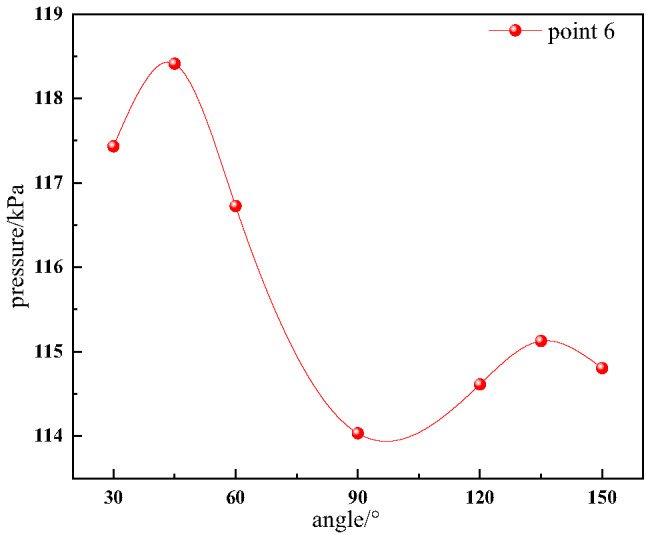
Evolution trends of the first pressure peak value with angle for measuring point 6.

As shown in Fig. 14, the pressure wave generated in the horizontal tunnel is affected by the dual influences of pressure diversion and pressure accumulation in the bifurcation structure as it propagates towards the bifurcation tunnel. When the bifurcation tunnel angle is 30° or 45°, the diversion effect of the bifurcation tunnel is stronger, the pressure wave tends to propagate towards the bifurcation tunnel, and the pressure accumulation effect in the bifurcation tunnel leads to a gradual increase in the pressure peak value. When the bifurcation tunnel angle is 60°, 120°, 135°, or 150°, the diversion effect of the bifurcation tunnel is relatively stronger. Specifically the pressure wave tends to propagate towards the bifurcation tunnel, and the pressure peak value decreases. However, it then begins to increase due to the dominant pressure accumulation effect in the bifurcation tunnel.

To clearly observe the impact of bifurcation tunnel angle on the attenuation law of explosion shock waves inside the bifurcation tunnel, measuring points 4 and 11 were selected for analysis. Let P4 be the first pressure peak value at measuring point 4 and P11 be the first pressure peak value at measuring point 11. The overpressure attenuation coefficient αc of the shock wave in the bifurcation tunnel is as follows^34^:

10 $${\alpha _{\mathrm{C}}}={P_{11}} \div {P_4}$$

**Fig. 15:**
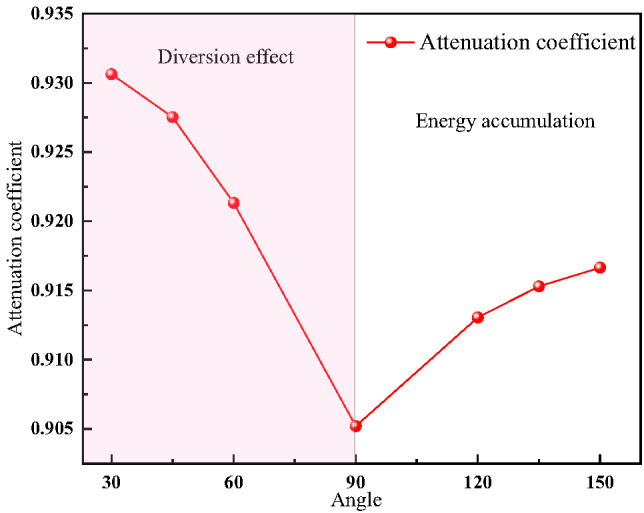
Evolution trends of the first pressure peak value attenuation coefficient with angle.

The attenuation coefficients of the first pressure peak value in the bifurcation tunnel with different bifurcation angles were calculated, as shown in Fig. 15. The attenuation coefficients of the first pressure peak value in the bifurcation tunnel generally show a trend of first decreasing and then increasing with the increase in bifurcation angle, ranging from 0.905 to 0.935.

As shown in Fig. 15, the attenuation coefficient is affected by the dual influences of the diversion effect and the energy accumulation effect of the bifurcation structure. Therefore, the attenuation coefficient does not monotonically change with the increase in angle. When the angle is small, the diversion effect of the bifurcation structure is dominant. As the angle increases, the diversion effect of the pressure in the bifurcation tunnel gradually decreases, resulting in a gradual decrease in the attenuation coefficient. When the bifurcation angle is an obtuse angle, the energy accumulation effect of the bifurcation structure is dominant. Therefore, as the angle increases, the energy accumulation effect gradually strengthens, resulting in an increase in the attenuation coefficient.

(2) Explosion pressure time history curve

**Fig. 16:**
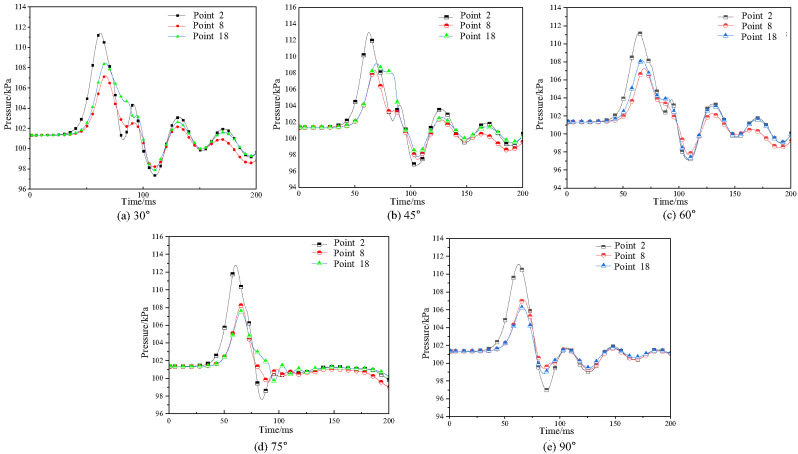
Explosion pressure time history curves in the bifurcation tunnel under different bifurcation angles.

To analyze the propagation path of gas explosion shock waves, measuring points 2, 8, and 18 in the bifurcation tunnel were used as research objects. The explosion pressure time history curves inside the horizontal tunnel and bifurcation tunnel (30°, 45°, 60°, 75°, 90°) were obtained, as shown in Fig. 16.

As shown in Fig. 16, as the bifurcation tunnel angle increases, the explosion pressure time history curves at each measuring point inside the tunnel show a similar trend, first increasing and then decreasing. Under different bifurcation tunnel conditions, the peak explosion pressure at measuring point 2 shows little variation. The explosion pressure peak value at measuring point 8 gradually increases with the increase in bifurcation tunnel angle, with the increase gradually decreasing. The explosion pressure peak value at measuring point 18 gradually decreases with the increase in bifurcation tunnel angle, with the decrease gradually decreasing. The above results indicate that the larger the bifurcation tunnel angle, the smaller the reduction of explosion pressure in the horizontal tunnel. Simultaneously, the arrival times of shock waves at all measurement points increased with greater bifurcation angles, indicating a reduction in propagation velocity. This delay stemmed from increased path lengths and enhanced interactions at larger bifurcation angles. The most pronounced delay occurred at measurement point 18, demonstrating that shock waves entering the bifurcated branch experienced greater attenuation compared to those in the main tunnel.

It can also be observed that pressure trends are similar across different angles. This is because when the bifurcation angle is small, the pressure wave’s diversion effect is significant, causing pressure to accumulate within the bifurcated tunnel and rise with increasing distance. Conversely, when the angle is large, although the initial diversion effect weakens, leading to an initial pressure drop, subsequent pressure increases occur due to enhanced reflection from tunnel walls, accumulated energy effects, and pressure wave superposition. Despite angular variations, the system’s initial conditions and primary influencing factors remain fundamentally unchanged. Consequently, the overall driving forces and constraints governing pressure generation, development, and variation exhibit similarity across angles. This results in comparable pressure values and trends at different angles, particularly during specific wave propagation stages-such as the early peak phase or reflection superposition phase-where geometric differences are masked by the inherent characteristics of wave propagation.

(3) Explosion pressure and peak value

**Fig. 17:**
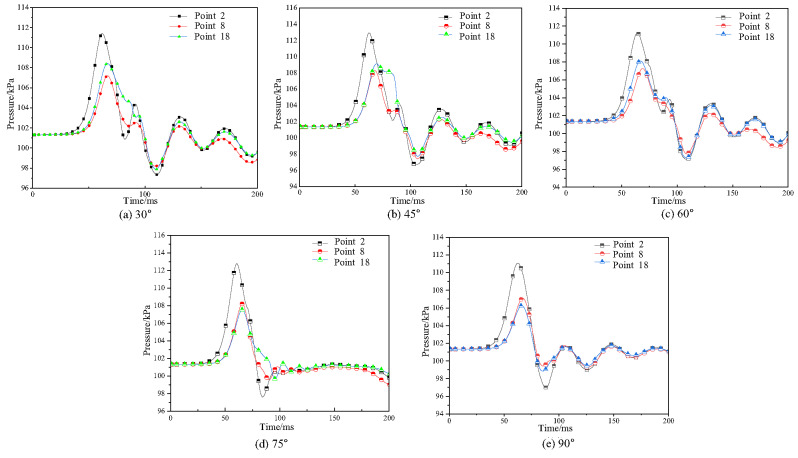
Explosion pressure distribution under different bifurcation tunnel angles.

As shown in Fig. 17, as the bifurcation tunnel angle increases, the larger the bifurcation angle of the bifurcation tunnel inside the tunnel, the smaller the diversion effect on the straight tunnel, and the two mutually affect each other. The attenuation of shock waves in bifurcation tunnels is greater than in straight tunnels^35^. When there is a bifurcation tunnel, the time required for the pressure wave to reach the end face of the bifurcation tunnel and form a reflected wave is short, allowing it to quickly converge and superimpose with the pressure wave in the main tunnel, causing the pressure and temperature of the unburned gas behind the wave to rise rapidly, thereby enhancing the combustion chemical reaction rate and ultimately increasing the explosion intensity.

**Fig. 18:**
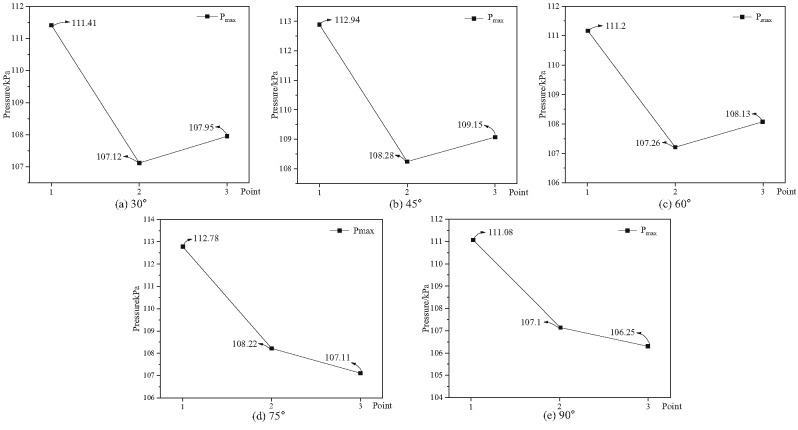
Explosion peak curves of bifurcation tunnels under different angles.

**Fig. 19:**
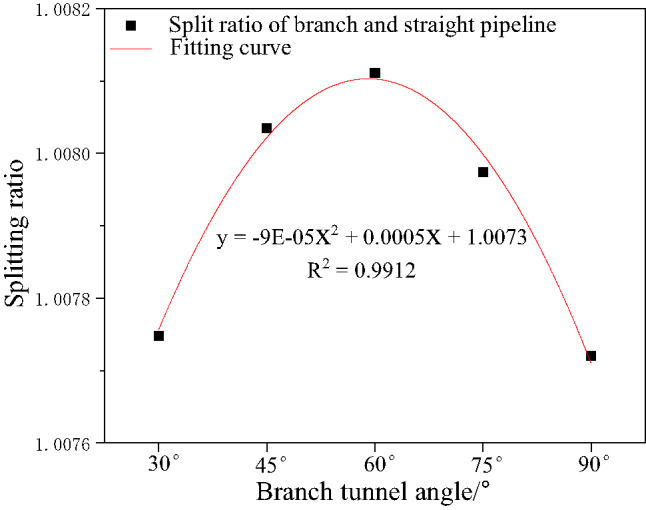
Trend of diversion ratio under different bifurcation angles.

The explosion pressure peak values at measuring points 2, 8, and 18 were extracted from Fig. 17, and the explosion peak curves of bifurcation tunnels under different bifurcation angles were obtained, as shown in Fig. 18. The diversion ratio of bifurcation tunnels under different bifurcation angles was also calculated, as shown in Fig. 19.

Figure 19 illustrates the variation trend of the flow distribution ratio in bifurcated tunnels at different bifurcation angles. This figure quantifies the energy distribution pattern of explosive shock waves within bifurcated tunnel structures, visually demonstrating how the bifurcation angle influences the selection of shock wave propagation paths and the distribution of pressure energy. Through the diversion ratio parameter, the guiding effect of bifurcated structures on shock waves can be clearly defined, thereby revealing the regulatory mechanism of geometric structures on wave propagation behavior. Shock wave attenuation in bifurcated tunnels is significantly greater than in straight tunnels. As the bifurcation angle increases, methane explosion pressure gradually dissipates from the horizontal tunnel. The greater the branching angle, the weaker the diversion effect on the straight tunnel. When the branching angle ranges from 30 to 60 degrees, the diversion coefficient between the branched tunnel and the straight tunnel falls between 1.00775 and 1.0081. However, when the branching angle reaches 60 to 90 degrees, this coefficient ranges from 1.00771 to 1.0081.

The diversion ratio results are directly linked to the previous wave attenuation findings: changes in the diversion ratio reflect the distribution of energy between the divergent tunnel and the main tunnel, and this energy distribution directly influences the degree of shock wave attenuation along each path. As the diversion ratio increases, more energy enters the divergent tunnel, potentially leading to accelerated pressure decay in the main tunnel-consistent with the trend in attenuation coefficients shown in Figs. 10 and 15. simultaneously, the non-monotonic variation of the diversion ratio with angle explains the initial decrease followed by an increase in pressure peaks observed in Figs. 13 and 14, demonstrating that the bifurcated structure influences wave attenuation behavior through energy redistribution.

## Conclusions

1. Through experimental and numerical simulation verification, it was found that when the mesh size is 2 cm, the average absolute error and average relative error between the simulated values and experimental values are the smallest, at 1.54 kPa and 1.47%, respectively. The size of the air domain does not affect the numerical simulation results. The analysis suggests that the errors between the numerical simulation data and experimental data mainly arise from the accuracy and sensitivity of the pressure sensors, heat loss from the walls, and the roughness of the tunnel walls.
2. By simulating the propagation of shock waves in single tunnels with different bending angles, it was found that as the angle of the horizontal tunnel increases, each measuring point in the tunnel first shows a multi-peak trend, followed by a larger peak value, with the peak pressure showing an initial increase, then a decrease, followed by another increase. Bending tunnels reduce the attenuation of gas explosion pressure, and the larger the bending angle, the slower the attenuation of the gas explosion.
3. Simulation studies on shock wave propagation in tunnels with different branching angles indicate that when the branching angle is 30° or 45°, the initial pressure peak within the branching tunnel increases with distance. However, when the branching angle is 60°, 120°, 135°, or 150°, this peak first decreases and then increases with distance. The shock wave attenuation coefficient in bifurcated tunnels exhibits a trend of decreasing then increasing with increasing bifurcation angle, and the overall attenuation is greater than that in straight tunnels, in diverging tunnels with angles ranging from 60° to 150°, the attenuation coefficient varies between 0.905 and 0.935, approximately 5% to 10% higher than measurements in straight tunnels. This indicates significantly greater energy dissipation within diverging structures.

## Data Availability

The datasets used and/or analysed during the current study available from the corresponding author on reasonable request.
